# The versatility of multi-state models for the analysis of longitudinal data with unobservable features

**DOI:** 10.1007/s10985-012-9236-2

**Published:** 2012-12-06

**Authors:** Vernon T. Farewell, Brian D. M. Tom

**Affiliations:** MRC Biostatistics Unit, Institute of Public Health, Robinson Way, Cambridge, CB2 0SR UK

**Keywords:** Causal inference, Classification uncertainty, Informative missing data, Multi-state models, Time dependent explanatory variables

## Abstract

Multi-state models provide a convenient statistical framework for a wide variety of medical applications characterized by multiple events and longitudinal data. We illustrate this through four examples. The potential value of the incorporation of unobserved or partially observed states is highlighted. In addition, joint modelling of multiple processes is illustrated with application to potentially informative loss to follow-up, mis-measured or missclassified data and causal inference.

## Introduction

Ross Prentice’s work has influenced and inspired many generations of academic and applied statisticians. His contributions to event history analysis have been notable, both in terms of the breadth of topics covered and the insights made. His book with Jack Kalbfleisch on “The Statistical Analysis of Failure Time Data” (Kalbfleisch and Prentice 2002) is a classic, and a must have text for those wanting a thorough grounding in methods for the analysis of failure time data. His ability to quickly identify and grasp the fundamental nature of problems and the issues arising in applications is impressive, resulting in many important methodological and applied works. Throughout over 40 years of statistical research, he has shown the power and flexibility of survival analysis techniques to successfully tackle a range of problems.

The aim of this current paper is to demonstrate that the multi-state modelling approach provides a convenient framework for handling a wide variety of medical applications characterized by multiple events and longitudinal data. While not explicitly formulated in terms of multi-state models, the work of Ross Prentice presented in his 1981 pioneering paper on multivariate failure times (Prentice et al. 1981) informs much of the model building presented here. If multiple events can be observed in longitudinal follow-up, then consideration must be given to the re-formulation of time-to-event models after the occurrence of events. Baseline hazards, the definition of time-dependent explanatory variables, the choice of time scale, the incorporation of history and the interpretability of formulations may all need to be carefully examined.

Prentice et al. (1981) is just one example of Ross Prentice’s work where the practical application of the latest development in statistical methodology, there Cox’s (1972) relative risk regression model, was carefully implemented, further developed where necessary and then used to clearly illustrate the potential of the method. We wish to use a number of applications to demonstrate the same potential and versatility of multi-state models.

Aalen et al. (2008) highlights the value of a process point of view and, in particular, the use of multi-state models for handling the temporal aspects of longitudinal data and for reflecting the inherent structures of particular applications. In the four examples examined in this paper, we will specifically demonstrate the potential value of the incorporation of partially observable states, and the use of multi-state models as an approach to joint modelling, including that of loss to follow-up, for dealing with mis-measured or misclassified data, and to aid causal inference. The proposed multi-state models or their estimation will all involve the handling of some unobserved information about one or more of state occupancy, transition times or random effects. While illustrative results will be provided for all examples, our focus will be on model formulation. Details of parameter estimation will not be provided, although references will be given.

Our first example will focus on joint modelling of two related processes and a sensitivity analysis related to potentially informative censoring.

## Semi-competing risks and informative observation

In biostatistics and epidemiology, Prentice et al. (1978) convincingly argued that the framing of standard competing risks problems, where occurrence of a competing event precludes the occurrence of other events, in particular the event of interest, in terms of conceptual or latent failure times is inappropriate and should be avoided. Instead, they advocate that the formulation of competing risks problems in these areas should be in terms of multi-state processes, since reference to conceptual times is avoided and focus instead is placed on the cause-specific hazard functions (i.e. the transition intensity functions), which are the basic estimable/identifiable quantities. Although the nomenclature “multi-state processes” was not explicitly used in Prentice et al. (1978), their inherent structure is clearly evident in the paper.

In a variation to the competing risks problem, semi-competing risks (Fine et al. 2001) refer to the situation when, in longitudinal follow-up of subjects, there are two events of interest, a terminal and a non-terminal one. The terminal event can censor the non-terminal one but not vice-versa. Here more information about event times are available than in the standard competing risks setting as data beyond the first event (if not terminal) are collected. Again we would argue that developing models based on latent event times should be avoided and multi-state models are preferable, if not advisable. In this section we describe a multi-state modelling approach (Siannis et al. 2007) for analysing data of this type that arose in the Whitehall II study, a large epidemiological study of British civil servants (CS).

### A multi-state model for the Whitehall II study

The Whitehall longitudinal cohort included 10,308 men and women aged 35–55 years who were recruited between 1985 and 1988 (Marmot et al. 1991). Data used here were from four follow-up study phases, the last ending in 1999. One outcome of interest is the occurrence of serious coronary heart disease (CHD) events. These events can be either fatal ($F$) or non-fatal (*NF*). A link to the National Health Service Central Registry provided accurate information on the date and cause of death for all CS who died. Thus complete information is available on $F$ events but data on *NF* events are subject to interval censoring when a CS is lost to follow-up.

A key question of interest was the relationship between the grade of employment ‘level’ of a civil servant and CHD risk. It was recognized however that loss to follow-up may be linked to health status and thus that censoring could be informative. Therefore it was important to understand how robust any findings related to the risks associated with civil service grade were to informative censoring, and a sensitivity analysis to address this was desired.

If we regard the two expressions of CHD (*NF* and $F$) as two separate, but perhaps dependent, events, then a model for two processes, *NF* and $F$, operating simultaneously is needed. During the observation period, each CS can experience none, one or even both of the events, the latter being possible only if an *NF* event happens first. *NF* events can also occur after the CS has been lost to follow-up (LTF) for the *NF*-process. In this case the *NF* event will be unobserved. No loss to follow-up is possible for the mortality process.

**Fig. 1:**
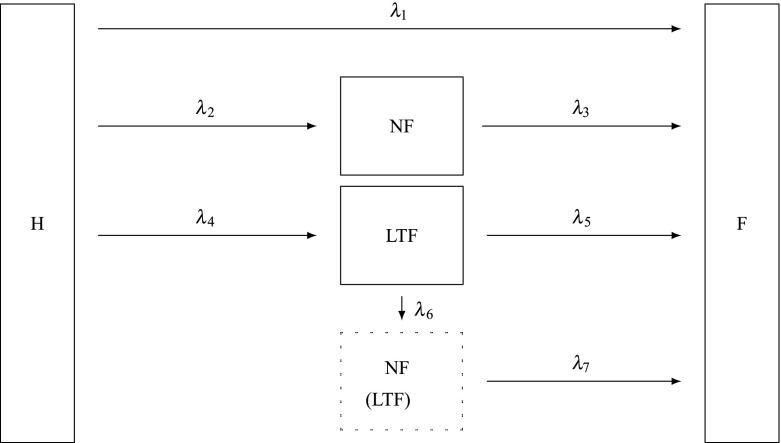
Whitehall II multi-state model (*H* healthy, *NF* non-fatal CHD event, *LTF* lost to follow-up, *F* fatal CHD event)

To model the two processes, the multi-state model presented in Fig. 1 can be used. This model, with five possible states, allows all possible combinations of events to be represented. Although we have complete information for the $F$-process, we have no information from a CS after they are LTF to the *NF*-process until the end of follow-up for the $F$-process (either death or end of study). In particular, we have no information on *NF* events that happen during that period of time. However, we allow for the possibility of such an event through inclusion of an unobservable state. The five states in the model are the healthy state, ‘H’, the fatal state, ‘F’, which is absorbing, a state, ‘NF’, to represent an observed non-fatal event, a state, ‘LTF’, to represent loss- to-follow-up for the *NF*-process, and the unobservable state, ‘NF(LTF)’, that represents an *NF* event experienced after the CS is lost-to-follow-up for the *NF*-process.

Note that for simplicity, we have described this multi-state model without regard to the non-CHD related deaths. In the subsequent analysis of the data, these deaths are treated as arising from a competing risk for the CS in the Whitehall II Study. Thus the estimated CHD-related hazard functions in our model are actually cause-specific hazards in a slightly more complex model. As in a typical competing risks analysis, we estimate these hazard functions by treating death from non-CHD related deaths as independently right censored.

Likelihood estimation for the model in Fig. 1 is outlined in Siannis et al. (2007), where the transition rates are assumed to have a proportional hazards structure with a Weibull baseline hazard function, with the time from entry into the study, denoted by $t,$ taken as the time scale. However, for this model to be identifiable, at least two assumptions, that relate to the baseline transition intensity functions $\lambda _5(t),\,\lambda _6(t)$ and $\lambda _7(t),$ are required. The model fitting will be dependent on these assumptions, and hence they need to be plausible and subject to some level of sensitivity analysis.

The first assumption is that $\lambda _3(t) = \lambda _7(t).$ This implies that whether or not a subject is censored, the risks of death after having an *NF* event are the same. The second is that

$$\begin{aligned} \frac{\lambda _1(t)}{\lambda _2(t)} = k \frac{\lambda _5(t)}{\lambda _6(t)}, \end{aligned}$$

which implies that, although the risk, or hazard, associated with having either an $F$ or *NF* event could be different after a subject is LTF than before, the ratios of these hazards before and after LTF are assumed proportional, and, when $k=1,$ equal.

The proposed assumptions are motivated by the particular multi-state model and are not data-driven. They provide a means to identify the transition rates introduced because an unobservable state has been added to the model. The parameter $k$ cannot be estimated and can serve only as a sensitivity parameter. However, it may be valuable to compare predictions from these alternative multi-state models with those from dynamic predictions made through alternative methods such as landmarking (van Houwelingen and Putter 2008).

### Illustrative Whitehall II results

As mentioned earlier, the role of employment grade is of primary interest. A relevant test of significance can be based on the multi-state model. A further simplifying assumption can be made that the explanatory variable effects related to all transitions to the $F$ state are the same. Then, to carry out the significance test, the multi-state models with the grade effect allowed to vary across the different hazards (six df for grade, two for each freely varying transition rate) and the grade effect completely removed (zero df for grade) are fitted. A likelihood ratio (LR) test to compare the two models leads to the test statistic $LR=142.68,$ which, when compared to the $\chi ^{2}$ distribution with six df, is highly significant ($p<0.0001$). This demonstrates a significant role for grade level.

**Fig. 2:**
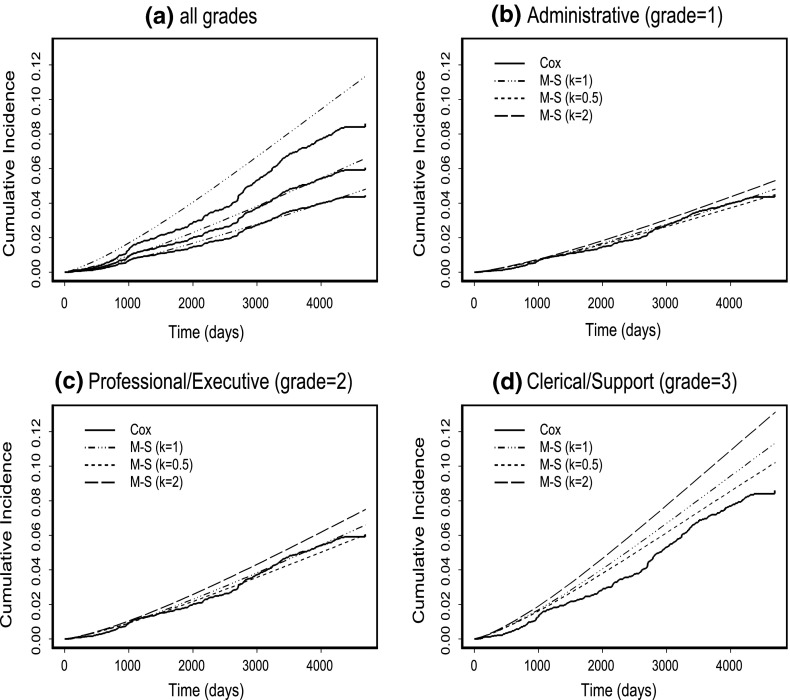
Comparison of time-to-first event Cox cumulative incidence curves with those from sensitivity analyses for the multi-state (M-S) model by different grade levels and restricted to males aged 45–49 years. **a** Curves are estimated with k=1

The possible inadequacies of analyses based on the time-to-first event, either *NF* or $F,$ and the time-to-NF event in the competing risks setting where we ignore the $F$-process subsequent to occurrence of the *NF* event, were also a motivating factor for the semi-competing risks multi-state model developed since the follow-up of subjects after an *NF* event can and should be incorporated. To illustrate the differences in results obtained from performing a time-to-first event Cox regression analysis and the proposed multi-state analysis, we present the cumulative incidence functions from both these analyses by grade level, restricted to males aged 45–49 years. Figure 2a presents the results for all grade levels but with the multi-state results restricted to $k=1.$ The remaining figures (Fig. 2b–d) present for each grade level in turn, the Cox results and the multi-state results from sensitivity analyses with $k=0.5, 1$ and $2.$ It can be seen that the multi-state model estimates more of an increased risk for males associated with grade 3 than does the time-to-first event analysis. The differences for the other grade levels are much less marked.

### Remarks

Standard methods, such as those based on Cox’s relative risk regression model, can provide analyses of the (semi-)competing risks separately and investigate, to an extent, the relationship between the risk of the terminal and non-terminal event through the use of time-dependent covariates. However such methods do not provide a comprehensive joint analysis for semi-competing risks data, as they do with standard competing risks data. An analysis of time-to-first event is particularly problematic using standard methods since some potentially informative data must be ignored.

The multi-state model presented here, with an unobservable state, provides another approach to the analysis of semi-competing risks data. While two model-based assumptions are necessary to ensure model identifiability, the model does provide a structure that clearly reflects the observed data and allows full use to be made of them. Explanatory variables are easily incorporated and estimation of various quantities of interest is possible. Importantly, the association between the risks and potentially informative LTF is introduced in a rather general way.

The next section presents another example of joint modelling through a multi-state structure but where the focus is more specifically on the relationship between processes and where a specific form of measurement error is a particular concern. It, and all our subsequent examples, will derive from the analysis of data from psoriatic arthritis (PsA) patients.

## An expanded multi-state model for joint modelling

Time-dependent explanatory variables arise commonly in medical applications. Cox’s seminal paper (Cox 1972) provided a practical approach to their incorporation in time-to-event models but, when observation is not made in continuous time, many pragmatic issues must be addressed. Notably important is the effect of infrequently updating time-dependent variables which typically leads to attenuation of regression effects in the standard survival analysis context (Raboud et al. 1993; Andersen and Liestøl 2003). Because of the intermittent nature of recording of explanatory variable information, the modelling of such data is akin to the the modelling of survival data subject to covariate measurement error to which Prentice has made significant contributions (Prentice 1982; Pepe et al. 1989; Wang et al. 1997).

To illustrate the usefulness of multi-state models in this regard, we consider a longitudinal investigation of quality of life in 600 PsA patients. PsA is an inflammatory arthritis associated with the skin disease psoriasis. Due to the combination of joint and skin manifestations of the disease, PsA can have a substantial impact on patient function, well being and health-related quality of life (Gladman et al. 2007; Sokoll and Helliwell 2001; Mease 2009). At the University of Toronto PsA Clinic, patients are scheduled to be seen at approximately 6-month intervals and physical functional disability, as measured by the health assessment questionnaire (HAQ) (Fries et al. 1980), has been assessed on an approximately annual basis (median time between HAQ administrations 1.08 years; inter-quartile range (1.00, 1.42) years) since 1993.

The focus of this investigation was on the relationship between physical functioning and other factors. For example, sex as a time-independent variable and the number of permanently damaged joints, a time-dependent measure of disease progression, were of interest. However, physical functioning is known to correlate significantly with current disease activity, reflected in the number of tender or effused joints at any point in time. Thus there is interest in controlling for disease activity when studying the relationship between functional disability and other explanatory variables.

To reflect this situation, consider that an outcome (e.g. HAQ) at any continuous time $t,\,Y(t),$ can take $m$ possible ordered states from the set $\{1,\ldots , m\},$ where transitions are allowed in both directions between adjacent states but direct transitions are not permitted between non-adjacent states. This constraint on the transitions is in continuous time, and therefore is not as restrictive as it first appears since an observed transition between non-adjacent states over two successive visits in the data would necessarily have been via the unobserved “adjacent” states in the period between these visits. In addition, we assume, without any real loss of generality, that there is one potentially rapidly fluctuating time-dependent ordinal variable (e.g. disease activity), denoted by $W(t),$ which is allowed to take $n$ possible distinct values from the set $\{1,\ldots , n\},$ and that there are two other explanatory variables which are of direct interest; one a less changeable time-dependent explanatory variable, $X(t)$ (i.e. damage), and the other a time-independent variable, $Z$ (i.e. sex). For ease of exposition, we describe a model for the $i$th of $N$ subjects.

**Fig. 3:**
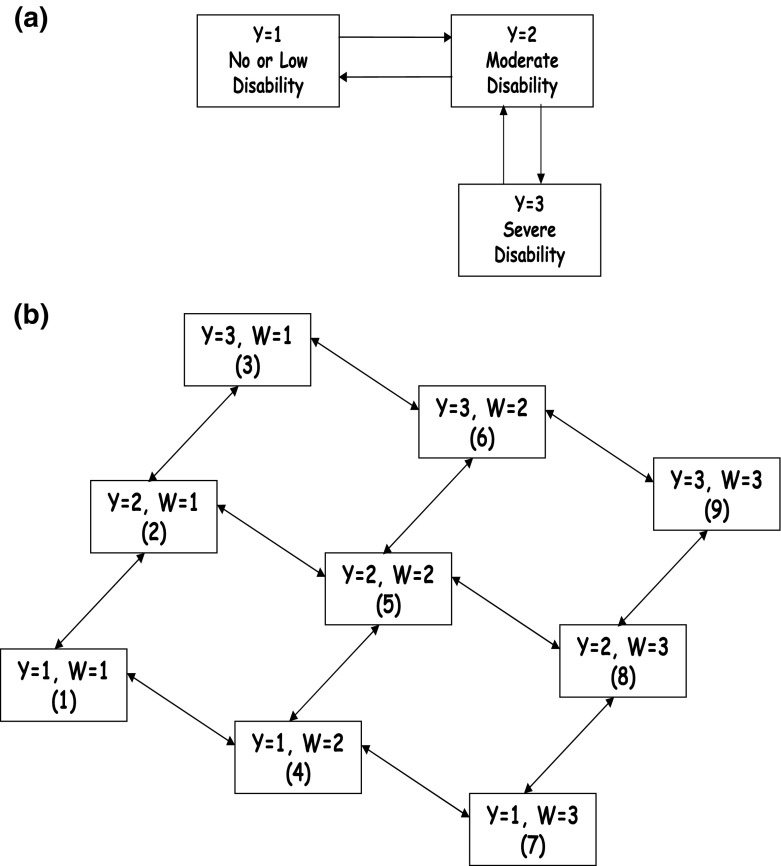
Three-state and nine-state multi-state diagrams for functional disability states of $Y$ and for combined states of $[Y,W]$ (*Y* functional disability, *W* disease activity)

Assume the outcome, $Y_i(t),$ and the predictable vector, $\mathbf{{V}}_i(t) = (W_i(t),X_i(t),Z_i)^{\text{ T}},$ of the three explanatory variables for this $i$th subject are recorded intermittently at $p_i+1$ observation/visit times $0=t_{i0} < t_{i1} <\cdots < t_{ip_i}$ (assumed ignorable (Grüger et al. 1991)), and that the evolution of $Y_i(t),$ can be described in terms of a multi-state model with the transition rate from state $j$ to state $k$ (denoted by $j \rightarrow k$) defined by a proportional rate of the form:

1 $$\begin{aligned} \lambda _{ijk}(t; \mathbf{{v}}_i(t)) = \left\{ \begin{array}{ll} \lambda _{0jk}(t)\exp (\alpha _{jk}^{\text{ T}}\mathbf{{w}}_{Ii}(t)+ \beta _{jk}x_i(t)+\gamma _{jk}z_i)&\quad \text{ if} |j-k|=1 \\ 0&\quad \text{ otherwise,} \end{array} \right. \end{aligned}$$

where $\lambda _{0jk}(t)$ represents the baseline rate function for the $j \rightarrow k$ transition, $\alpha _{jk}=(\alpha _{2jk}, \ldots , \alpha _{njk})^{\text{ T}}$ is the vector of regression coefficients associated with the vector of dummy variables, $\mathbf{{w}}_{Ii}(t) = (I(w_i(t)=2), \ldots , I(w_i(t)=n))^{\text{ T}},$ and $\beta _{jk}$ and $\gamma _{jk}$ are the regression parameters associated with the two variables of interest, $x(t)$ and $z,$ respectively. Figure 3a illustrates such a multi-state model for $Y(t)$ representing three functional disability states.

Because the Toronto PsA HAQ data are based on annual assessments, i.e. represent panel data based on intermittent observation, fitting of (1) is difficult, without at least an additional assumption, since the times at which time-dependent variables change values are not observed. The most common simplifying assumption is that the transition rate matrix, $\varLambda (t),$ is piecewise-constant. This is usually achieved by approximating time-dependent variables as piecewise-constant functions, where these variables are assumed constant between the times/visits for which they are available, and by specifying the baseline rate functions either to be time homogeneous (i.e. constant) or piecewise-constant.

For explanatory variables that change rather slowly over time, as assumed for $X(t),$ the assumption of piecewise constancy between visits may be a reasonable approximation. However for some variables, such as the more rapidly varying $W(t),$ this assumption may be highly questionable. Subsequently, we will refer to the simplified multi-state model obtained by making the piecewise-constancy assumption for both $X(t)$ and $W(t)$ as the misspecified model.

To recognise explicitly the intermittent observation of $W(t),$ the structure of the multi-state model given in (1) can be expanded by jointly modelling the outcome of interest, $Y(t),$ and the time-dependent variable of concern, $W(t),$ through a larger multi-state model with $m \times n$ states. However, in this new combined process, $[Y,W](t),$ it is important to retain the distinction between $Y(t)$ as the outcome of primary interest and $W(t)$ as a *predictable* time-varying explanatory variable given in (1). To do this it is necessary to assume that $W(t+h) \bot \!\!\! \bot Y(t)| W(t), \forall h>0,$ where $A \bot \!\!\! \bot B | C$ means $A$ is conditionally independent of $B$ given $C$ (Didelez 2007; Dawid 1979). However note that this does not imply that $W(t+h) \bot \!\!\! \bot Y(t+h) | [Y,W](t).$

The states in the new model are defined by the unique combinations of the states of $Y(t)$ and the levels of $W(t).$ This is illustrated in Fig. 3b for $n=3$ levels of $W(t).$ For this structure, standard multi-state software (e.g. the msm package (Jackson 2011) in R (R Development Core Team 2010)), that accommodates panel data, can be used with interval censored observation of transition times between the states of $Y(t)$ and the intermittent observation of the explanatory variable, $W(t).$ This approach, although more general in principle, can be made consistent with (1) through constraints on the regression coefficients and baseline rates.

Formally, the correspondence between the multi-state model (1) and the expanded multi-state representation can be made explicit when any two expanded states are denoted by $(j,r) \equiv j_r = j+m(r - 1)$ and $(k,s) \equiv k_s = k+m(s-1)$ ($(j,r) \ne (k,s)$) and where the transition rates between these states of the joint process $[Y,W](t)$ are written as

2 $$\begin{aligned} \lambda _{ij_rk_s}(t;x_i(t),z_i)=\left\{ \begin{array}{ll} \lambda _{0j_rk_s}(t)\exp (\beta _{j_rk_s}x_i(t)+\gamma _{j_rk_s}z_i)&\quad \text{ if} |j_r-k_s|=1 \text{ or} m \\ 0&\quad \text{ otherwise.} \end{array} \right. \end{aligned}$$

As outlined in more detail in Tom and Farewell (2011), to ensure correspondence between models, it is necessary to place constraints on the baseline rates, $\lambda _{0j_rk_s}(t),$ and the regression parameters, $\beta _{j_rk_s}$ and $\gamma _{j_rk_s}.$ These constraints will force the regression coefficients in the rates for all upward transitions between the same two $Y$ states to be same and, similarly, for downward transitions. Additionally, transition rates, specified by the baseline transitions rates and the regression coefficients, between the same two $W$ states are constrained to be identical. These latter constraints are required since model (1) specifies the distribution of $(Y(t)|W(t-),X(t-),Z)$ and thus we need to model the “marginal” distribution of $(W(t-)|X(t-),Z)$ to get at the joint distribution. Note that in (2) we do not allow the two sub-processes $Y(t)$ and $W(t)$ to change states at the same time but, as mentioned earlier, this is not much of a restriction when modelling in continuous time. We refer to the multi-state model corresponding to (2) with the aforementioned imposed constraints as the expanded model.

The constraints placed on the regression parameters in (2) allow them to be interpreted in exactly the same way as the corresponding regression parameters in the simpler multi-state model defined by (1). The baseline transition rates corresponding to movements between states of the $Y(t)$ sub-process are unconstrained to model the dependence of $Y(t)$ process on $W(t-).$ Finally note that we have permitted explanatory variables to have modifying effects on the baseline transition rates relating to movement between the levels of the $W(t)$ sub-process. This is practically important if these variables confound the relationship between $W(t)$ and $Y(t)$ and, more generally, mimics the usual regression formulation where no assumption of independence between explanatory variables is made.

### Illustrative HAQ results

Table 1 presents selected results from fitting two multi-state models to the Toronto PsA data. The first fits the misspecified three-state model for physical functioning (HAQ) alone with updating of both disease activity and damage only at the times of clinic visits. The second fits the expanded multi-state model of Fig. 3b where the fluctuating nature of disease activity is reflected. Both models included age and arthritis duration as explanatory variables but results are only presented for sex and damaged joint count, along with the estimated disease activity effects on fatigue transitions derived from the logarithms of ratios of baseline rates for the expanded model and from the regression coefficients for the misspecified three-state model.

**Table 1:** Multi-state modelling results for sex and damage effects on transitions between disability states, controlling for the levels of disease activity, as well as age and arthritis duration (results not show)

Variable	Disability transition	Multi-state representations	
		Misspecified model	Expanded model
		Estimate (95 % CI)	Estimate (95 % CI)
Sex			
Male vs. female	$1 \rightarrow 2$	$-0.7578 (-1.0350, -0.4801)$	$-0.6444 (-0.9315, -0.3573)$
Male vs. female	$2 \rightarrow 3$	$-0.1977 (-0.6329, 0.2376)$	$-0.2483 (-0.6911, 0.1945)$
Male vs. female	$2 \rightarrow 1$	$0.0931 (-0.1749, 0.3610)$	$0.1640 (-0.1110, 0.4391)$
Male vs. female	$3 \rightarrow 2$	$0.1506 (-0.2506, 0.5518)$	$0.07219 (-0.3393, 0.4837)$
Number of damaged joints			
	$1 \rightarrow 2$	$0.0106 (-0.0019, 0.0231)$	$0.0103 (-0.0025, 0.0230)$
	$2 \rightarrow 3$	$0.0036 (-0.0112, 0.0183)$	$0.0086 (-0.0070, 0.0242)$
	$2 \rightarrow 1$	$-0.0166 (-0.0273, -0.0058)$	$-0.0201 (-0.0307, -0.0095)$
	$3 \rightarrow 2$	$-0.0116 (-0.0244, 0.0013)$	$-0.0168 (-0.0302, -0.0033)$
Number of active joints			
[1,5] vs. 0	$1 \rightarrow 2$	0.4892 (0.1774, 0.8009)	1.0283 (0.4972, 1.5594)
[1,5] vs. 0	$2 \rightarrow 3$	$0.2943 (-0.3530, 0.9416)$	$1.1029 (-0.2906, 2.3491)$
[1,5] vs. 0	$2 \rightarrow 1$	$0.1004 (-0.2576, 0.4584)$	$0.1429 (-0.3331, 0.6188)$
[1,5] vs. 0	$3 \rightarrow 2$	$-0.1865 (-0.8610, 0.4879)$	$-0.2847 (-1.2181, 0.6486)$
$>$5 vs. 0	$1 \rightarrow 2$	$0.7924 (0.4269, 1.1580)$	$1.6063 (1.1409, 2.0716)$
$>$5 vs. 0	$2 \rightarrow 3$	0.7286 (0.1158, 1.3410)	1.7995 (0.6943, 2.9047)
$>$5 vs. 0	$2 \rightarrow 1$	$-0.0045 (-0.3605, 0.3515)$	$-0.9484 (-1.4376, -0.4592)$
$>$5 vs. 0	$3 \rightarrow 2$	$-0.5073 (-1.1490, 0.1344)$	$-1.0239 (-1.7621, -0.2858)$

While attenuation of activity effects from this misspecified model is quite marked and would be expected based on earlier work (Raboud et al. 1993; Andersen and Liestøl 2003), this will not necessarily be the case for other explanatory variables. For example, consider the effect of gender on the $1\rightarrow 2$ transition corresponding to an upward move from none or low disability to moderate disability and the effect of the number of clinically damaged joints on the $3 \rightarrow 2$ transition corresponding to a downward move from high disability to moderate disability. For the former, even though it is a time-independent variable, we observe an 18 % inflation in the absolute effect size for being male compared to female when we use the misspecified model instead of the expanded model, with the statistical significance of this effect becoming stronger ($Z$-statistic = 5.36 vs. 4.40). For the latter, we observe a 31 % reduction in the absolute effect of a one clinically damaged joint increase on the instantaneous transition from high disability to moderate disability, with a somewhat larger $p$-value ($p$ = 0.077 vs. 0.014). The possibility of both attenuation and strengthening of effects has also been demonstrated in simulation studies (Tom and Farewell 2011).

### Remarks

Intermittent observation of time-varying explanatory variables is common and, to a large extent, pragmatic assumptions are made to deal with this. However, many regression analyses of longitudinal data can be configured in terms of a multi-state model. If panel data is available for the estimation of the model and, in addition, there is one key time-varying explanatory variable which is reasonably represented by an ordinal variable, then the use of an expanded multi-state model may be useful. Minimally, it could act as the basis for a sensitivity analysis with respect to the potential impact of the more commonly made simplifying assumptions with respect to the pattern of the explanatory variable over time. Moreover an advantage, as opposed to other joint modelling approaches to coping with measurement error, is that it allows for the uncertainty in this key explanatory variable without explicitly having to model the rapid changes between visits.

The next section returns to the introduction into a multi-state model of unobservable or partially observable states and illustrates how the incorporation of a misclassification structure can allow the fitting of such a model.

## A model for remission in psoriatic arthritis

As indicated in the previous section, a basic measure of disease activity for patients with psoriatic arthritis is the number of tender or effused joints. These counts may vary considerably across clinic visits and zero counts are not uncommon. However, an extended period of time with no active joints is expected for patients who are in remission and the identification of factors associated with remission is of interest. For example, in Gladman et al. (2001), patients were defined to be in remission if they were observed to have no evidence of disease activity for at least three consecutive clinic visits.

In this situation, and more generally for time-to-event analysis of any event which is operationally defined by some condition being true for a prolonged period of time, defining the time to an event is problematic. Multi-state models offer an alternative to the use of simple but possibly inappropriate definitions of the time to an event.

### A three-state model

To begin, we regard remission as a conceptual disease state with those patients not in remission assumed to be in an active disease state. Operationally however, we link remission to the active joint count as follows. If a patient has one or more active joints at some time point, then their disease state is active. Thus any patient in remission must have no active joints and this can be regarded as a structural zero. If a patient has had three consecutive clinic visits when no active joints were observed, then the patient is regarded as being in remission at the last clinic visit during this period. Note that this consistent observation is not taken to define remission but is taken to *confirm* that the patient has entered remission at some point prior to the third visit. Given the intended pattern of clinic visits, three consecutive clinic visits will usually take place over a period of at least a year, and must be greater than 6 months given the definition of visits for the purposes of data collection. A patient with no active joints at a clinic visit that is not preceded by at least two other such visits may be in remission but it cannot be confirmed. They may also have active disease with the observed zero joint count being a sampling zero.

**Fig. 4:**
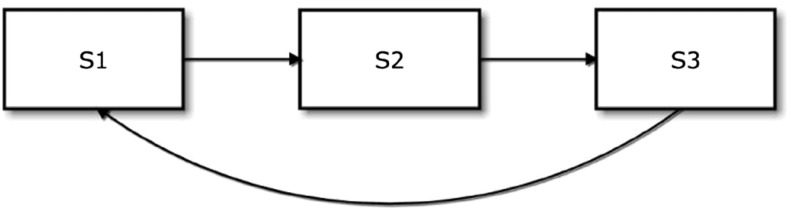
Three-state model for remission in PsA (*S1* active disease, *S2* early stage of remission, *S3* established stage of remission)

A three-state model that reflects this structure is given in Fig. 4. Suppose that $S(t)$ represents the state for a patient at time $t,$ where $t$ denotes the years since diagnosis of PsA. In this model, state $1\,(S(t)=S_1$) corresponds to active disease, state $2\,(S(t)=S_2$) corresponds to the *early* stage of remission and state $3\,(S(t)=S_3$) corresponds to an *established* stage of remission. It should be noted that a patient in either $S_2$ or $S_3$ is regarded as in remission. These are conceptual, not observed, states that are introduced in order to give all remissions some duration. This is achieved by the further assumption that patients in $S_2$ can only move to $S_3.$ Transitions back to active disease, $S_1,$ can only occur from $S_3.$

This model can be further specified by three transition rates. Associated with the $S_1 \rightarrow S_2$ transition, the first is the instantaneous rate of progression to $S_2,$ conditionally on occupying $S_1$ at $t$:

3 $$\begin{aligned} \lambda _{12}\left(t; \mathbf{{x}}(t)\right)&= \lambda _{012}(t)\exp \left(\mathbf{{\beta }}_{12}^{\text{ T}}\mathbf{{x}}(t)\right), \end{aligned}$$

where $\lambda _{012}(t)$ is a baseline transition rate, $\mathbf{{x}}(t)$ represents a vector of (possibly time-dependent) explanatory variables and $\mathbf{{\beta }}_{12}$ is the corresponding unknown vector of regression coefficients. Typically, this transition rate, (3), into $S_{2}$ would be the transition of greatest interest as it would provide a model for the distribution of time to remission from an active disease state.

A transition rate for the $S_3 \rightarrow S_1$ transition, $\lambda _{31}(t ; \mathbf{{x}}(t)),$ can be defined analogously and corresponds to a model for transitions from remission back to active disease. The final transition, $S_2 \rightarrow S_3,$ will have a rate of the same form but this conceptual transition is unlikely to be of clinical interest.

In fitting the model, the uncertainty regarding a patient’s state at some time point can be made explicit through the introduction of misclassification probabilities. Suppose that $S(t)=r$ ($r=S_1, S_2, S_3$) represents the true underlying state of a patient at time $t,$ and $O(t)=s$ ($s=O_1, O_2, O_3$) represents the corresponding observed state at time $t$ based on active joint counts. Specifically, let $O_1$ denote the observation of a non-zero active joint count at a visit, $O_2$ denote the observation of a zero active joint count at a visit not preceded by at least two other such visits, and $O_3$ denote the observation of the third or subsequent zero active joint counts in a sequence of such visits. Then, based on the assumptions about active joint counts outlined earlier and the assumption that the misclassification probabilities are independent of time $t,$ we can specify $\Pr \{O(t)=s \mid S(t)=r\}$ as follows:

1. $\Pr (O_1 \mid S_1)= 1- \Pr (O_2 \mid S_1) ,\,\Pr (O_2 \mid S_1)=\text{ logit}\,^{-1}(\mathbf{{\gamma }}^{\text{ T}}\mathbf{{z}}),\,\Pr (O_3 \mid S_1) = 0,$ where **z** is a vector of explanatory variables that might be informative about the true state of the patient at time $t,$ and $\mathbf{{\gamma }}$ is the corresponding unknown vector of regression coefficients;
2. $$\Pr (O_1 \mid S_2) = 0,\,\Pr (O_2 \mid S_2) = 1,\,\Pr (O_3 \mid S_2) = 0$$;
3. $$\Pr (O_1 \mid S_3) =0,\,\Pr (O_2 \mid S_3) =0,\,\Pr (O_3 \mid S_3) =1.$$

Essentially, only $S_1$ is allowed to be misclassified. Thus, at some time point $t,$ a patient with a clinic visit and an associated zero active joint count not preceded by at least two other such visits can either be in active disease ($S_1$) or be in early stage of remission ($S_2$). A logistic model is used to investigate the influence of explanatory variables $\mathbf{{z}}$ on $\Pr (O_2 \mid S_1).$ Specifically, for illustration, a binary explanatory variable, $Z,$ will be included. It is coded one for the first visit with a zero joint count not preceded by any other such visits and zero for visits with a zero active joint count only preceded by one such visit. The variable is defined only for visits corresponding to observed state $O_{2}.$

Multi-state models with misclassification have been discussed in Jackson et al. (2003) and can be fit using the msm R package under the assumption that transition rates are constant (i.e., time-independent) or piece-wise constant over time. Because the $(O_1, O_2, O_3)$ classification is only observed at clinic visits, the maximum likelihood estimation is based on the panel data arising from this intermittent observation pattern and the interval censoring option of the msm package must be used.

For comparison, two other approaches can be used to define remission in the PsA data. The first treats a patient as in remission at the end of a time period with three consecutive clinic visits with no active joints, while in the second approach, a patient is considered in remission at the beginning of such a period. Following Farewell and Su (2011), the three-state model can be denoted as Model A and the two other possibilities outlined here as Models B and C respectively.

### Illustrative remission result

A detailed analysis of data from 790 patients entering the Toronto PsA Clinic in the years 1973–2006 can be found in Farewell and Su (2011). The time scale $t$ is taken to be time since diagnosis of PsA. Typically patients will come to clinic at some time after diagnosis so likelihood estimation must incorporate left truncation of observation and this is also implemented in the R package msm. After exploratory analyses, baseline rates were taken to be piecewise constant in the two intervals $[0, 15]$ and $[15, 40].$

Models B and C would both have only two alternating states, corresponding to $S_{1}$ and $S_{3}$ in the three-state model, and no misclassification. In Model B, it is assumed that patients were in remission only at the third of three or more consecutive clinic visits with zero active joint counts (either from enrollment or following a visit with a non-zero active joint count). That is, a transition to remission had occurred between the second and third clinic visits of a time period with three consecutive visits with zero active joint counts. In Model C, it is assumed that remission had occurred by the first visit of such a period and after the previous visit at which a non-zero count was observed.

Two explanatory variables were included in the transition rate models for $\lambda _{12}(t)$ and $\lambda _{31}(t).$ Regression coefficients and standard errors for these variables for Models A, B and C are given in Table 2.

**Table 2:** Regression coefficients and 95 % confidence intervals for remission models

	Sex	Age at PsA diagnosis
Model A		
$S_1 \rightarrow S_2$	0.61 (0.34, 0.88)	0.18 (0.05, 0.31)
$S_3 \rightarrow S_1$	$-$0.20 ($-$0.52, 0.13)	0.46 (0.30, 0.62)
Model B		
$S_1 \rightarrow S_2$	0.75 (0.45, 1.05)	0.06 ($-$0.09, 0.20)
$S_3 \rightarrow S_1$	$-$0.32 ($-$0.64, 0.00)	$-$0.07 ($-$0.24, 0.09)
Model C		
$S_1 \rightarrow S_2$	0.83 (0.54, 1.12)	0.10 ($-$0.04, 0.24)
$S_3 \rightarrow S_1$	$-$0.21 ($-$0.54, 0.10)	0.08 ($-$0.09, 0.24)

The models can also be compared when used to estimate the total length of stay in various states over a fixed time period. If a period of 40 years after PsA diagnosis is considered, then the estimated time with active disease is 30.6, 32.5 and 29.5 years in Models A, B and C, respectively. Correspondingly, the time in remission (time spent in either $S_2$ or $S_3$ in Model A) for the same patients follows the reverse ordering. The results for female patients who were 35 years old at PsA diagnosis are different with the estimated time with active disease being 34.6, 37.0 and 35.5 years in Models A, B and C, respectively.

With the most conservative approach of defining remission, Model B always gives the least time spent in remission. Model A allows the possibility that, at the visits with zero active joint counts but not preceded by at least two other such visits, the patients were actually in early stage of remission. Therefore the estimated time spent in remission in Model A is expected to be longer than in Model B. In Model C, we allow the patients to be in remission exactly two clinic visits earlier than in Model B, which also makes the estimated time spent in remission longer. The ranking of the corresponding estimates from Models A and C will depend on specific scenarios. Model A allows a patient to possibly be in remission at visits with zero active joint counts but not preceded by at least two other such visits, regardless of the observed states at the following visits. In Model C, patients could be in remission as early as at the first visit of a sequence of three visits with zero counts. However, no possibility is given to remission at those one or two visits with zero active joint counts not preceded by at least two other such visits and immediately being followed by a visit with non-zero active joint count or reaching the study cut-off date. Thus a trade-off between these factors determines the estimated lengths of stay from Models A and C.

The fit of Model A is further characterized by the estimated misclassification probabilities. While $\Pr \left\{ O(t)=O_2 \mid S(t)= S_1, Z\right\}$ is directly modelled here, the quantity $\Pr \left\{ S(t)= S_1\mid O(t)=O_2, \mathbf{{x}}, Z \right\} ,$ corresponding to the probability that patients were actually in active disease when observed at clinic visits with zero active joint counts not preceded by at least two other such visits, given the explanatory variables, is more relevant. The latter probability can be calculated via Bayes’ rule if $\Pr \left\{ S(t)=S_1 \mid \mathbf{{x}} \right\}$ and $\Pr \left\{ S(t)=S_2 \mid \mathbf{{x}} \right\}$ are approximated by the proportions of estimated total length of stay in $S_1$ and $S_2$ for the time periods $t \in [0, 15)$ and $t \in [15, 40],$ given the explanatory variables.

This calculation, for male patients diagnosed at age 35, gives misclassification probabilities, $\Pr \left\{ S(t)= S_1\mid O(t)=O_2 \right\} ,$ of 0.938 and 0.322 for the first ($Z=1$) and second consecutive visits ($Z=0$) with zero counts during the first 15 years of disease and values of 0.810 and 0.118 subsequently. For females, the comparable numbers are 0.965 and 0.468 in the first time period and 0.887 and 0.199 in the second. As expected, the misclassification probabilities at the second visit with a zero count are much smaller than at the first.

### Remarks

The benefit of using this three-state model for remission is that it avoids ad hoc definitions of time to remission. However, to do this a “hypothetical” transition must be introduced to allow a plausible time in remission. Nevertheless, because misclassification is included in the model formulation, the assignment of observed states known to be problematic is avoided. Minimally, the use of the three-state model could be seen as a check on the robustness of findings from more simplistic approaches which might be adequate for some purposes and is another example of a multi-state model providing an approach to sensitivity analyses.

In the next section, we consider the joint modelling of many processes. This will involve the fitting of many correlated multi-state models, and in the particular application, each of these models will represent two separate processes as well. In addition, we will consider the extent to which causal arguments can be linked to results from estimation of these models.

## Correlated multi-state processes and causality

Previous sections have dealt with a variety of multi-state models but, in all cases, observed transitions for an individual have been assumed independent of those for all other individuals. However, this independence assumption is neither necessary nor sensible for some potential applications. We illustrate this with reference to an analysis of individual joint data from PsA patients.

Disease progression in PsA, as with rheumatoid arthritis (RA), is often taken to be reflected in the accumulation and severity of damaged joints. The damage process is an irreversible one, therefore once a joint is damaged it will remain so. Disease activity, on the other hand, is a reversible process, and is reflected in part by joints being described as either tender only or effused (joint swelling with or without tenderness); with the latter representing a more severe level of activity than the former. For both PsA and RA there is a strong belief amongst clinicians that active inflammation results in or causes joint damage. A number of research groups have repeatedly shown an association between disease activity and progression to damage. For example, in an investigation of the link between activity and damage in PsA, based on the Toronto PsA data (Bond and Farewell 2009), negative binomial regression models for the increase in the total damaged joint count between visits were fitted, with previous damage incorporated as a dynamic explanatory variable in the models to account for the within-patient correlation. Disease activity was initially included into these models both in terms of total active joint counts at clinic entry and as time-dependent explanatory variables, with the total joint counts updated at clinic visits. These models found that time varying activity (both effused and tender total joint counts) was associated with the progression of damage, but that activity variables at clinic entry were not, when their time varying counterparts were known.

Because total joint counts were used in these investigations, the relationships identified were essentially systemic. If we can examine the link between activity and damage at the individual joint level then this would further understanding of the biological processes and strengthen causal arguments. In addition, disease patterns, such as symmetry, can be investigated.

### A damage model for individual joints

There are 28 hand joints in total (excluding the wrists) and we consider a model for the 14 pairs of comparable joints on the left and right hands.

**Fig. 5:**
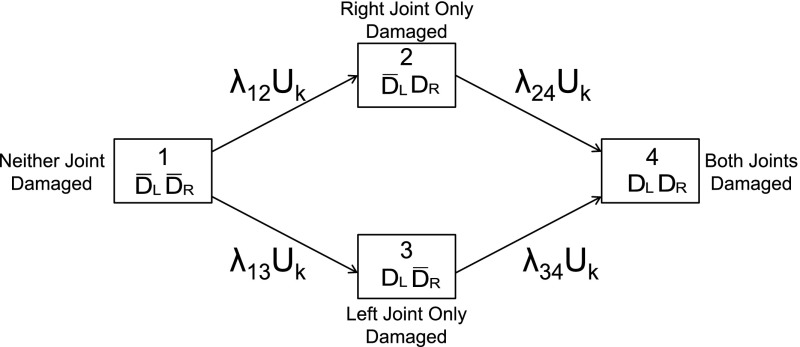
Diagram of the multi-state model for damage at a joint location, with random effect

A four-state model for damage in each of the 14 pairs of hand joints is depicted in Fig. 5. The four states of this multi-state model are defined as

- $S_{1}$: Damage in neither hand, $(\bar{D}_L,\bar{D}_R),$
- $S_{2}$: Damage in the right hand, $(\bar{D}_L, D_R),$
- $S_{3}$: Damage in the left hand, $(D_L, \bar{D}_R),$ and
- $S_{4}$: Damage in both hands, $(D_L, D_R).$

Note that this is a multi-state model at a specific joint location in both left and right hands.

Schweder (1970) introduced the concept of local (in)dependence between components of a composable finite Markov process. He felt that many studied phenomena can be realistically described by time-continuous finite Markov processes. If, in addition, the Markov process representing the phenomenon under study could be defined to be composable (i.e. represented as a vector of distinct sub-processes, whereby no two sub-processes or components can change state ‘simultaneously’), then (in)dependencies between sub-processes can be explicitly expressed through the transition rates of the original Markov process. The model in Fig. 4 is composable, comprising separate damage sub-processes for the left and right hands at the specific joint location, and local dependencies between the sub-processes will be reflected in relationships between the transition rates of this multi-state model. In addition, this model does not allow transitions directly from $S_{1}$ to $S_{4}.$ This is a necessary constraint to ensure composability but is not very restrictive for models with transitions in continuous time. Transitions between $S_{2}$ and $S_{3}$ are not allowed since damage is irreversible. Furthermore, because the 14 multi-state processes within a patient should be more similar than across patients, we introduce subject-specific random effects into the model. These act multiplicatively on the baseline transition rates of the 517 patients. The random effects, $U_k,\ k=1,\ldots ,517,$ are assumed to be distributed as independent Gamma random variables with unit mean and variance $\theta$ which we denote by $U_{k} \sim \text{ Gamma}(1/\theta , 1/\theta ).$ This multi-state model is a generalization of Schweder’s model to incorporate random effects and is similar to the one proposed by Cook et al. (2004) for clustered progressive multi-state processes and is in the same spirit as earlier work done by Self and Prentice (1986) for multivariate failure time data. Nevertheless, this model still allows us to examine local dependencies through the transition rates.

To investigate the relationship between damage and the dynamic course of disease activity at the individual joint (location) level we define binary indicators of joint-level activity, $A_{L}(t), A_{R}(t)$ for the left and right joints respectively in a pair at time $t.$ Here, we make no distinction between activity in the form of tenderness and effusion. However, where a joint makes a transition into a state of damage, we are interested in assessing a possible dose–response relationship between joint activity and the rate at which damage occurs. Thus we also define dynamic binary indicators for joint-level tenderness, $T_{L}(t)$ and $T_{R}(t),$ and joint-level effusion (with or without tenderness but usually tender), $E_{L}(t)$ and $E_{R}(t),$ for the left ($L$) and right ($R$) hands at time $t.$ Hence, assuming that no previous damage has occurred to either joint ($S_{1}$), models for the transition rates out of $S_{1}$ are given by:

4 $$\begin{aligned} \lambda ^{(l)}_{12k}(t)&= u_{k}\lambda ^{(l)}_{012}\text{ exp}\left(\alpha _{L12}A_{L}^{(l)}(t) +\tau _{R12}T_{ R}^{(l)}(t)+\varepsilon _{R12}E_{R}^{(l)}(t)\right) \nonumber \\ \lambda ^{(l)}_{13k}(t)&= u_{k}\lambda ^{(l)}_{013}\text{ exp}\left(\alpha _{R13}A_{R}^{(l)}(t) +\tau _{L13}T_{ L}^{(l )}(t)+\varepsilon _{L13}E_{L}^{(l)}(t)\right). \end{aligned}$$

We further assume that the baseline transition rates are constrained to be the same across the 14 hand-joint locations. That is, $\lambda ^{(l)}_{012}=\lambda _{012}$ and $\lambda ^{(l)}_{013}=\lambda _{013},\,\forall l.$ For simplicity, we do not explicitly denote the dependence of the rates on explanatory variables in the left hand side of the defining equations.

When forming models for the transition rates into $S_{4},$ we choose not to include information (in the form of explanatory variables) on the activity process in the opposite (damaged) joint at time $t$ because of the dominant effect of symmetrical damage to be discussed later. Hence, our models for the transition rates into $S_{4}$ are given by:

5 $$\begin{aligned} \lambda ^{(l)}_{24k}(t)&= u_{k}\lambda _{024}\text{ exp}\left(\tau _{L24}T_{L}^{(l)}(t) +\varepsilon _{L24}E_{L}^{(l)}(t)\right) \nonumber \\ \lambda ^{(l)}_{34k}(t)&= u_{k}\lambda _{034}\text{ exp}\left(\tau _{R34}T_{R}^{(l)}(t) +\varepsilon _{R34}E_{R}^{ (l)}(t)\right) \end{aligned}$$

and, once again, we assume that the baseline transition rates are constrained to be the same across the 14 joint locations.

Here, we focus on the relationship between observed transitions in the damage process and the value of these activity variables (represented by activity or joint tenderness and swelling on both the left and right hands) at the *last* clinic visit. That is, we assume $A_{L}(t),\,A_{R}(t),\,T_L(t),\,T_R(t),\,E_L(t)$ and $E_R(t)$ to be piecewise constant between clinic visits. As is done in Sect. 3, this assumption could be relaxed and is further discussed in O’Keeffe et al. (2011). We additionally assume that these activity variables can only change ‘state’ immediately after the time of a clinic visit when damage information becomes available.

It is ‘biologically’ conceivable that this model, given by (4) and (5), can be further constrained to allow $\alpha _{L12}=\alpha _{R13},\,\tau _{R12}=\tau _{L13},\, \tau _{L24}=\tau _{R34},$ and similarly, $\varepsilon _{R12}=\varepsilon _{L13},\,\varepsilon _{L24} =\varepsilon _{R34}.$ These constraints are used in the interest of parsimony.

We also include additional binary explanatory variables (for joints on the left and right hands) that indicate whether a joint was ever observed active (either swollen or tender) at the present visit or any of the previous protocol visits. We constrained the associated regression coefficients to be the same for transitions in the left and right hands and allowed only prior activity in the undamaged joint to have an effect on the corresponding transition into $S_{4}.$ These binary variables attempt to incorporate more of the history of disease activity (i.e. the persistence) in the hand joints of patients into the four-state damage model.

A symmetrical damage pattern in arthritis implies that the tendency for a joint at a specific location to become damaged is increased if the contralateral joint on the other hand is earlier damaged, and this applies at all joint locations. To investigate the extent of disease symmetry in the hands, it is useful to re-parameterize some of the baseline transition rates in terms of others. That is, we specify $\lambda _{024}^{(l)} = \lambda _{013}^{(l)}\exp (\gamma _{24})$ and $\lambda _{034}^{(l)}=\lambda _{012}^{(l)}\exp (\gamma _{34}),$ with no constraints placed on the baseline transition rates. Therefore a symmetric pattern would correspond to $\lambda _{012}^{(l)} < \lambda _{034}^{(l)}$ and $\lambda _{013}^{(l)} < \lambda _{024}^{(l)},$ or equivalently that $\gamma _{24}>0$ and $\gamma _{34}>0.$

Maximum likelihood estimation of the parameters of the four-state damage model can be implemented and details are given in O’Keeffe et al. (2011).

### Illustrative damage results

To illustrate the use of the four-state damage model, we use data extracted from 517 of the 790 patients in the Toronto PsA clinic who entered before January, 2007. These 517 patients correspond to those who at clinic entry had no clinical damage in any of the hand joints on either the left or right hands and therefore information on all these patients is comparable.

Based on these data, and with no explanatory variables introduced into the transition rate models, the parameters, $\gamma _{24}$ and $\gamma _{34}$ defined above to characterize symmetry are estimated to be 1.82 and 1.39 respectively with corresponding 95 % confidence intervals of (1.49, 2.14) and (1.01, 1.78). Both of these confidence intervals indicate substantial departures from zero to the right, and therefore present strong evidence for symmetry. This symmetry, in turn, implies that there are local dependencies in both direction between the two damage sub-processes (i.e. the left and the right) of the composable multi-state joint location process.

Table 3 highlights estimation results related to the explanatory variables in the multi-state model defined in Subsect. 5.1 to investigate the relationship between disease activity and damage progression. Seven patients were excluded due to missing information on disease activity. In this table ‘transitive’ joint refers to the joint undergoing the transition to a state of damage (i.e. to $S_{2},\,S_3$ or $S_4$) and ‘opposite’ joint refers to the same joint in the opposite hand. From this table, where there is no previous damage in either joint (i.e. the model is currently in $S_{1}$), we observe increases in the rates of transitions into a state of damage when there is current activity (in the form of both tenderness and effusion) and when there has been some past activity in the transitive joint, compared to when no activity has been seen. We note that effusion shows a larger positive effect on transition to damage than tenderness in this situation. Conversely, activity in the opposite joint appears not to significantly affect the transition rate to damage.

**Table 3:** Log-rate ratio and rate ratio (RR) estimates for activity at the individual joint level, together with associated 95 % confidence intervals (CI)

Effect on transition to damage	Estimate (95 % CI)	RR (95 % CI)
*No previous damage in either joint*		
Tenderness in the transitive joint	1.01 (0.72, 1.31)	2.76 (2.06, 3.70)
Effusion in the transitive joint	1.50 (1.22, 1.77)	4.47 (3.38, 5.90)
Activity in the opposite joint	0.17 ($-$0.10, 0.44)	1.18 (0.90, 1.55)
Transitive joint active in the past	0.76 (0.52, 1.00)	2.14 (1.68, 2.71)
Opposite joint active in the past	0.10 ($-$0.15, 0.35)	1.10 (0.86, 1.41)

Effect on transition to damage	Estimate (95 % CI)	RR (95 % CI)
*Opposite joint damaged*		
Tenderness in the transitive joint	0.81 (0.41, 1.20)	2.24 (1.51, 3.32)
Effusion in the transitive joint	0.78 (0.34, 1.23)	2.19 (1.40, 3.41)
Transitive joint active in the past	0.31 (0.01, 0.62)	1.37 (1.00, 1.86)

In the situation where the opposite joint is already damaged, we again see an increase in the transition to damage both where there is current tenderness and where there is current effusion as well as where past activity has occurred in the transitive joint. We note that the effects of tenderness and effusion are similar; we no longer see an apparent dose–response relationship for the covariates representing activity in the transitive joint. Presumably this is related to the additional effect of symmetrical joint damage.

The log-rate ratios of the six transitive association effects described previously, are all positive and large, with probable differential effects observed between the comparable transitions for tender only and the more severe effused (usually also tender) joints, ipsilaterally, where no damage has occurred to the opposite joint. The effects corresponding to effusion tend to be larger than the comparable ones for tender only, ipsilaterally, where no damage has occurred to the opposite joint. These six association effects are all the statistically significant ones found and the four corresponding to current activity suggest that the link between activity and damage is local/specific to the joint on the particular hand being considered. These four associations are what we would consider as a local dependence/influence of activity on damage at a joint. The two significant associations corresponding to past activity in the transitive joint may indicate that the persistence of activity is also important in predicting future damage progression. We do not observe any statistically significant association, or evidence of a possibly substantive effect, of having or not having activity in a joint on one hand with having or not having damage on the contralateral joint of the other hand.

### Causal inference

Schweder (1970) regarded his concept of local (in)dependence as a potential aid in addressing causal questions and it is closely linked to the concept of Granger causality (Granger 1969). We note that Granger causality was initially defined in the context of discrete time series, and local (in)dependence may be viewed somewhat as an extension of the Granger causality/non-causality concept to processes in continuous time. The asymmetry of the local independence concept makes it particularly attractive, as having one sub-process locally influencing a change in another, but the other not having any influence on the first, is precisely how we would like to characterize a causal effect of one sub-process on another. However, a one-sided local dependence relationship between two sub-processes of a composable Markov process is not sufficient to imply causation. Aalen (1987), when extending the concept, stressed that local (in)dependence was a dynamic statistical approach which, by incorporating time explicitly, offers a natural way to model potential causal relationships.

Mathematical formalization of the causality concept, in itself, is not enough to allow a causal relationship to be inferred from an observed association. From an epidemiological viewpoint, Hill (1965) discussed aspects of an association that should be considered when attempting to infer causation. These are known as the Bradford Hill criteria and are (i) strength of association; (ii) consistency; (iii) specificity; (iv) temporality; (v) biological gradient; (vi) biological plausibility; (vii) coherence; (viii) experimental evidence (when available); and (ix) analogy. Hill stressed that these are only ‘viewpoints’ to be considered however and are not necessary and/or sufficient conditions to declare causation from an observed association, although temporality is a necessary condition as a cause must precede its effect.

Analysis of the Toronto PsA data provided evidence of a symmetric pattern of joint damage. For a pair of joints at the same location in the two hands, local dependencies were identified in both directions between the damage sub-processes in the left and right hands. While these results represent an important finding, these local dependencies do not immediately warrant a causal explanation, as they do not produce a one-sided (local dependence) asymmetric relationship between the two damage sub-processes. It is quite plausible that the same underlying biological mechanism is driving these two damage sub-processes, although a robust biological explanation for damage symmetry has not yet been established.

However, the analyses also suggest (i) joint-specificity of the relationship between activity and damage; (ii) strong associations for the four statistically significant and biologically plausible local effects obtained; and, where no previous damage has occurred in either joint of a pair, (iii) a dose–response relationship of activity with damage at the joint level (i.e. a biological gradient). These relationships represent local dependence of activity on damage although the activity process is not formally modelled. Here the determination of asymmetry of local dependence relationships is not specifically addressed and is less critical because any influence of permanent damage on subsequent activity in a joint is of less clinical interest or, at least, represents a very different clinical question. However, as Aalen argues, the dynamic perspective of Schweder’s work may be most important and these analyses, because of the longitudinal nature of the data, do help to characterize the temporal relationship between activity and damage.

These results are also consistent with the majority of the Bradford Hill Criteria: specificity, strength of association, biological gradient, temporality and biological plausibility, at the joint level. Others such as consistency and analogy have been shown at the patient (systemic) level in other PsA populations and in RA populations respectively. Moreover our results do not, in any way that we know of, conflict with generally known facts regarding the biology and natural history of disease progression in PsA patients, thus suggesting coherence. Furthermore recent clinical trials on biologic agents have shown the effectiveness of these therapies in slowing the disease progression. These agents calm the inflammation of arthritis by inhibiting immunological pathways that trigger inflammatory response. Therefore these results do provide support for a putative causal relationship between activity and damage. Also, we believe that, from the perspective of both biological plausibility and temporal ordering, this demonstrated relationship at the joint level offers more support for a causal link than the relationships at the patient level seen in previous investigations.

### Remarks

The study of the progression of PsA using multi-state models provided an intuitive way of examining the disease process from a dynamic perspective and afforded a straightforward way to assess local (in)dependencies in dynamic processes. The introduction of random effects allowed a natural way to model correlated multi-state processes so that individual joint data could be appropriately examined. Thus, it is possible to consider the extent to which evidence of local dependence between the activity and damage processes, together with the Bradford Hill Criteria, permits a causal link between activity and damage in this observational setting. However as Prentice and Thomas (1987) writes:

‘$\ldots$ any reported associations from an observational study will be subject to some uncertainty concerning causality. Associations that are strong, that exhibit “regular” dose–response relationships, and that can be replicated in a range of study populations come, in time, to be regarded as causal.’

Therefore it is over time and with replication and mechanistic understanding that the link between activity and damage will truly be regarded as causal.

## Conclusion

In many ways multi-state models are the natural generalization of standard time- to-event analysis and therefore it would be expected that the power and flexibility to tackle a range of problems seen with survival analysis techniques to filter through or even be amplified in the multi-state setting. This is indeed the case and the availability of software to allow such models to be fitted has increased their use in recent times.

For the examples in Sects. 3 and 4, the use of the msm R package (Jackson et al. 2003) with its many options, made estimation of the models relatively straightforward. For the examples of Sects. 2 and 5, bespoke R code was written and this did involve considerable programming time. However, the availability of numerical optimisation routines in R, as in many statistical packages, meant that the primary effort is directed only at calculation of likelihood functions. In both examples, the task was considerably simplified because the multi-state models are progressive. With intermittent observation, reversible models may present considerable computational challenges unless simplifying assumptions are made.

Unlike in the standard failure time setting where interest is on a random variable (i.e. the time to experiencing the event of interest), the focus in the multi-state situation is on the modelling of stochastic processes. As a result of this process point of view, many of the features of longitudinally observed data can be clearly and realistically reflected, and issues and problems that arise can be addressed quite naturally. This flexibility to cope with problems arising from longitudinal data is exemplified in the Whitehall II application when dealing with informative loss to follow-up in a semi-competing risks setting and in the remission application when defining the occurrence of an event based on prolonged observation and potential misclassification of the event. In both these cases, a hypothetical or unobservable state was introduced to reflect, in a clinically meaningful way, the inherent structures of the data-sets. In contrast, attempts to apply standard survival analysis methodology to the time- to-first CHD event and to an ad hoc operational definition of remission may result in not only possibly unrealistic assumptions being made and the inefficient use of all the data, but in misleading inference. At the least the multi-state modelling approach provides an opportunity to check the findings from these more simplistic approaches and offers a credible approach for performing sensitivity analyses. However, even for the purpose of sensitivity analyses, it is important to recognize that the use of “plausible” assumptions to resolve non-identifiability should be viewed cautiously. The work of Molenberghs et al. (2008) illustrates this in the case of informative missingness.

The utility of multi-state models is also apparent in handling intermittently updated time-varying explanatory variables, especially those that may be internal covariates, and in analysing clustered processes. Here a joint modelling approach can be adopted as illustrated in our second and fourth applications. In these examples, the dynamic nature of both the outcome processes (i.e. functional disability and damage) and the activity process were apparent, with the activity process potentially carrying crucial information about the times of transitions. Therefore it was important to appropriately incorporate the activity process into the multi-state models’ structures. Additionally, correlation could naturally be handled through the dynamicity of the multi-state approach, by the incorporation of history of the processes or through introduction of random effects.

Through the use of dynamic concepts such as composability, local independence and local dependence, and with sound application of the Bradford Hill criteria, multi-state models provide a powerful methodology for tackling important causal questions as demonstrated with our application on individual joint damage in the hands.

We hope that, through the four applications presented, a convincing argument for the versatility of multi-state models has been made.

Ross Prentice’s statistical career has been remarkable and he is equally remarkable as a person. It was a great pleasure to be invited to contribute to this issue of *Lifetime Data Analysis* published in his honour.
